# Crosstalk Between *Staphylococcus aureus* and Innate Immunity: Focus on Immunometabolism

**DOI:** 10.3389/fimmu.2020.621750

**Published:** 2021-02-05

**Authors:** Christopher M. Horn, Tammy Kielian

**Affiliations:** Department of Pathology and Microbiology, University of Nebraska Medical Center, Omaha, NE, United States

**Keywords:** *Staphylococcus aureus*, biofilm, immunometabolism, macrophage, myeloid-derived suppressor cell, lactate

## Abstract

*Staphylococcus aureus* is a leading cause of bacterial infections globally in both healthcare and community settings. The success of this bacterium is the product of an expansive repertoire of virulence factors in combination with acquired antibiotic resistance and propensity for biofilm formation. *S. aureus* leverages these factors to adapt to and subvert the host immune response. With the burgeoning field of immunometabolism, it has become clear that the metabolic program of leukocytes dictates their inflammatory status and overall effectiveness in clearing an infection. The metabolic flexibility of *S. aureus* offers an inherent means by which the pathogen could manipulate the infection milieu to promote its survival. The exact metabolic pathways that *S. aureus* influences in leukocytes are not entirely understood, and more work is needed to understand how *S. aureus* co-opts leukocyte metabolism to gain an advantage. In this review, we discuss the current knowledge concerning how metabolic biases dictate the pro- vs. anti-inflammatory attributes of various innate immune populations, how *S. aureus* metabolism influences leukocyte activation, and compare this with other bacterial pathogens. A better understanding of the metabolic crosstalk between *S. aureus* and leukocytes may unveil novel therapeutic strategies to combat these devastating infections.

## Introduction

*Staphylococcus aureus* (*S. aureus*) is an opportunistic pathogen that colonizes approximately one-third of the human population and can cause invasive disease at an array of different sites throughout the body, including endocarditis, skin and soft tissue infection, bacteremia, pneumonia, osteomyelitis, and medical implant-associated infection (1, 2). The ability to successfully infect and persist in such a wide range of tissue niches is due to a number of characteristics that allow the bacterium to evade immune-mediated clearance. Such attributes include the production of various toxins, the acquisition of antibiotic resistance or tolerance, and the ability to form biofilm (3–6). Each of these factors, often in combination with one another, contribute to the ability of *S. aureus* to counteract immune effector mechanisms.

Throughout the course of an infection, both host and pathogen undergo substantial changes in their metabolic programs to facilitate the production of different effector molecules that aid in their respective goals (7, 8). In the case of leukocytes, this takes the form of regulating the production of cytokines, such as IL-1β. For *S. aureus*, metabolic reprogramming allows for the production of various leukocidins and lactate, among other virulence factors, that combat the immune system (9). As an infection progresses, nutrient concentrations within the tissue milieu can rapidly fluctuate as host and pathogen compete for the same extracellular energy sources (10, 11). The intrinsic metabolic flexibility of *S. aureus* allows it to quickly adapt to these evolving conditions to promote its survival (12, 13). While *S. aureus* may be able to overcome the depletion of specific nutrient sources, the leukocyte population may not be as flexible. As metabolism is inextricably linked to immune cell function, the intentional depletion and/or release of specific metabolites that can impair leukocyte microbicidal activity could represent a system by which the bacteria influences host cell metabolism to its benefit. Here we provide an overview of how *S. aureus* interacts with leukocytes at the metabolic level. We will focus on immunomodulatory metabolites and how they contribute to the crosstalk between host and pathogen during an infection, with a particular emphasis on *S. aureus* biofilm formation. We also provide examples of metabolic crosstalk between leukocytes and other bacterial species as further mechanisms to consider in the context of *S. aureus* infection.

## Immunometabolism

**Figure 1:**
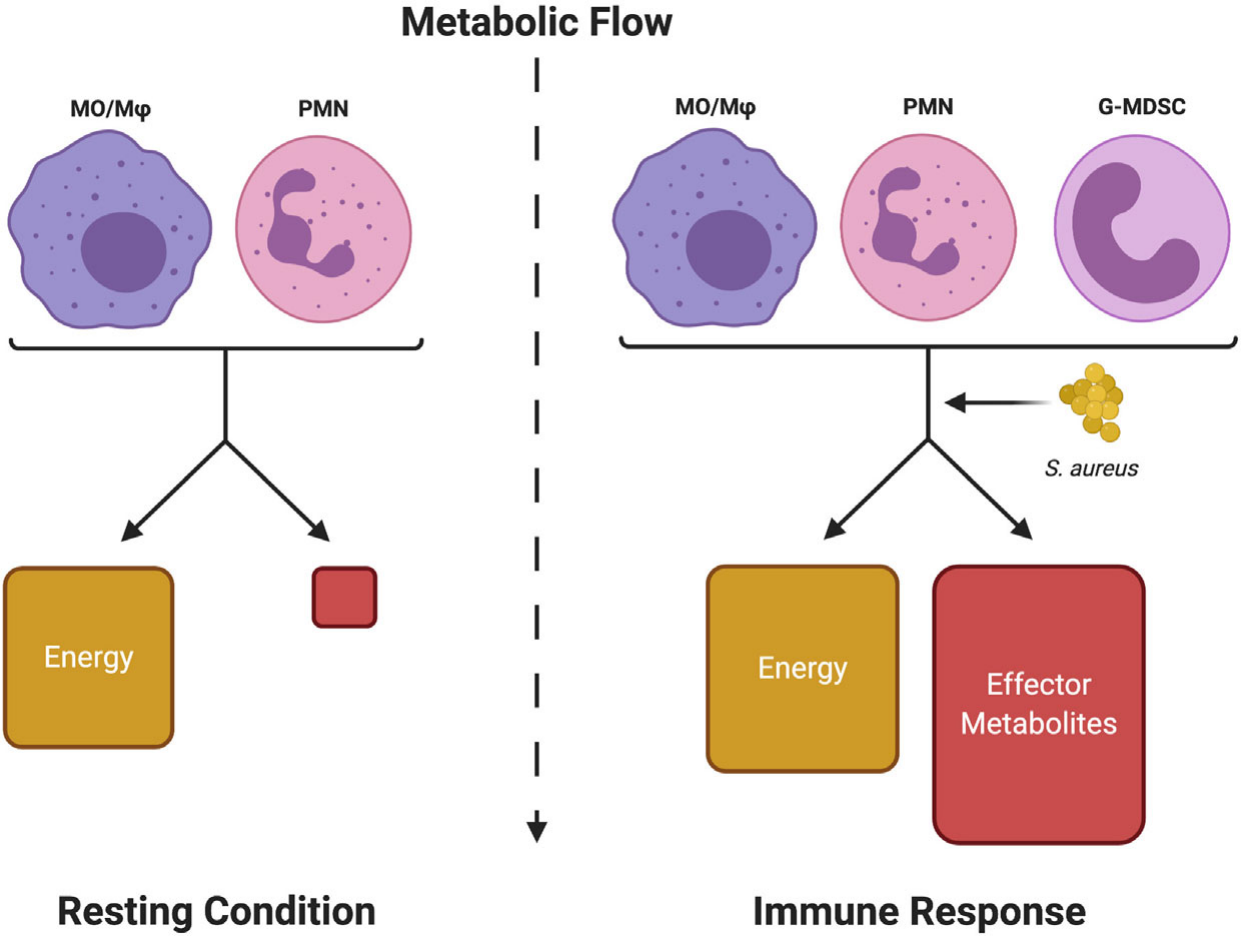
Metabolic shifts in leukocytes define resting from inflammatory states. Immune cells normally respire under resting conditions to provide sufficient energy (ATP) for survival. Upon activation, although energy production is somewhat increased, a significant portion of the metabolic flow is dedicated to producing metabolic intermediates (Effector Metabolites) that are used to generate inflammatory mediators (i.e. cytokines, inflammatory lipids, etc.) to promote leukocyte effector functions (Immune Response). *S. aureus*, either through competition for metabolic resources or by releasing immunomodulatory metabolites, can interfere with this process to disrupt a productive immune response. Monocyte (MO), macrophage (Mφ), neutrophil (PMN), and granulocytic myeloid-derived suppressor cell (G-MDSC). Figure created with BioRender.

The study of immunometabolism is focused on linking changes in metabolic programs to effector functions. Over the years, it has become apparent that activated leukocytes experience a shift in metabolism that regulates inflammatory mediator production (14–16). Activation usually coincides with a metabolic shift from the energy (ATP)-rich resting state to an increase in the production of effector metabolites necessary for biosynthetic processes for inflammatory effector function, such as fatty acid biosynthesis for prostaglandin production (**Figure 1**) (17–20). Since this observation was made, there has been considerable interest in defining metabolic programs that are characteristic of either the pro- or anti-inflammatory status of various leukocyte populations. Much of this fervor originated from the idea that immune cell function could potentially be orchestrated *via* the manipulation of the nutrient milieu or by therapeutically targeting specific metabolic pathways.

In the context of an infection, immunometabolism is only half of the story, since pathogens must also acquire essential nutrient sources for their survival (21). This leads to direct competition between the host and pathogen on a metabolic scale. Much like responding leukocytes, *S. aureus* must also undergo some degree of metabolic adaptation to counteract the effects of the immune response. Switching between alternative forms of metabolism is common in *S. aureus* due to its significant degree of metabolic plasticity (22–24). This flexibility greatly increases the chances that the bacteria will win the battle of attrition between host and pathogen for specific nutrient sources. If this ability to shift between different metabolic modes is compromised, it could drastically alter the landscape of an infection. For instance, work from our laboratory has established that *S. aureus* biofilm skews leukocytes towards an anti-inflammatory phenotype in a murine model of prosthetic joint infection (PJI) that is mediated, in part, by IL-10 production (25–28). Recent work demonstrated that the loss of an essential metabolic process in *S. aureus*, such as ATP synthesis (Δ*atpA*), significantly decreased the chronicity of biofilm infection by eliciting a heightened pro-inflammatory response (29). This is likely attributable to the respiratory defect in *S. aureus* Δ*atpA* that limits the energy required for virulence factor production (30), which subsequently led to increased leukocyte viability and pro-inflammatory activity (29). Another group has shown that metabolic adaptation of *Pseudomonas aeruginosa* is essential for establishing chronic biofilm infection in patients with debilitating diseases such as cystic fibrosis (CF). Host-adapted *P. aeruginosa* strains preferentially utilized the tricarboxylic acid (TCA) cycle to limit stress from reactive oxygen species (ROS) produced by leukocytes in the airway. This TCA metabolic bias in *P. aeruginosa* enhanced biofilm formation, which served to increase the chronicity of infection (31). How metabolic changes can influence the function of various leukocyte populations and an overview of critical *S. aureus* metabolic pathways in the host will be discussed in the following sections.

## Macrophage Metabolism

**Figure 2:**
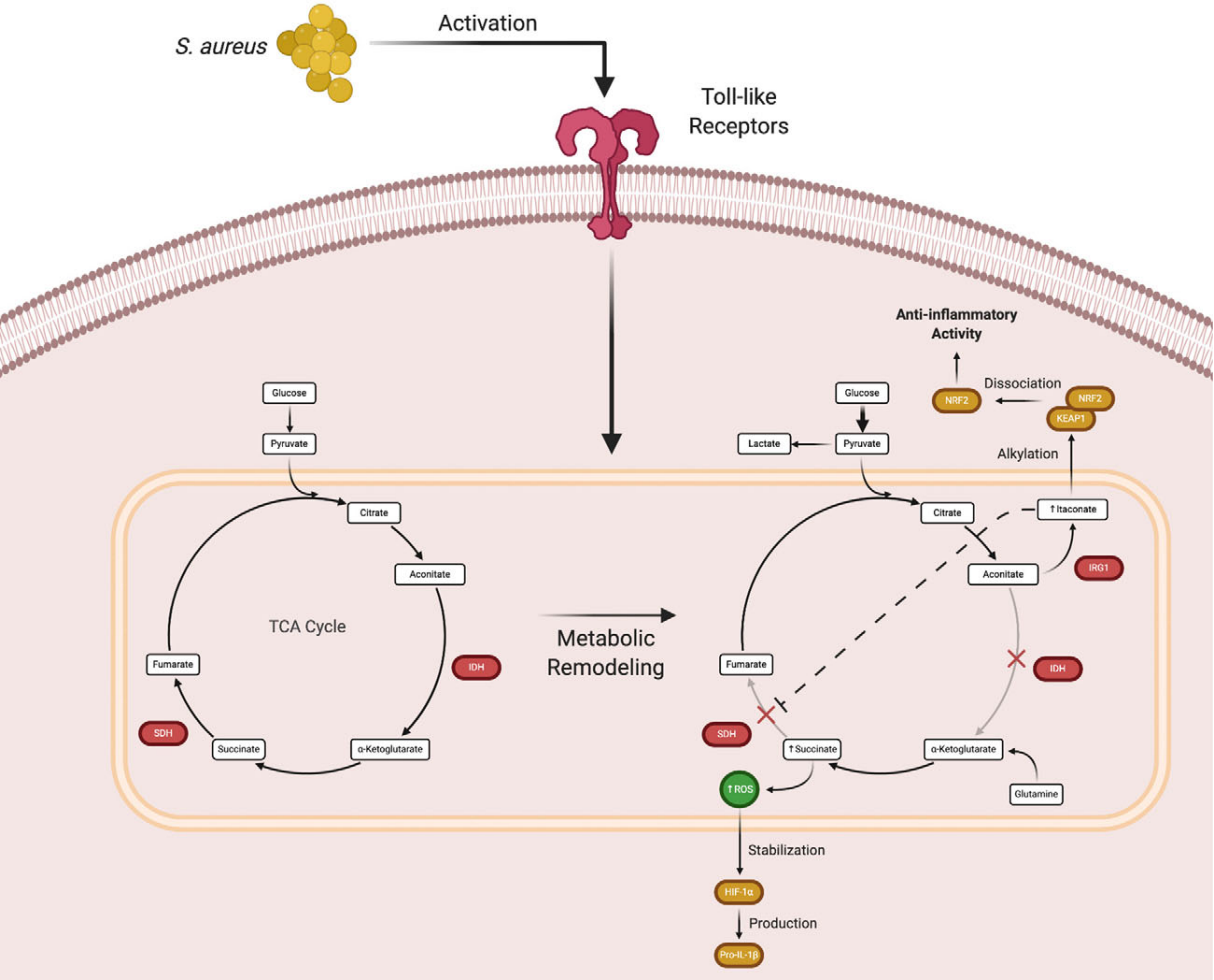
Macrophage metabolic remodeling during an immune response. Following Toll-like receptor (TLR) activation, macrophages undergo metabolic rewiring to promote inflammatory mediator production. The TCA cycle breaks at two points, isocitrate dehydrogenase (IDH) that causes the accumulation of aconitate, which is converted by immune responsive gene 1 (IRG1) to form itaconate, and succinate dehydrogenase (SDH) that leads to succinate accumulation. While this process normally augments proinflammatory activity, excess itaconate production eventually causes a shift to promote the expression of anti-inflammatory genes. Figure created with BioRender.

The macrophage is currently the prototypical cell studied in the immunometabolism field. Much progress has been made in characterizing how macrophages shift their metabolism after exposure to various stimuli *in vitro* and how this influences their effector functions. These experiments led to the discovery that pro- and anti-inflammatory polarized macrophages have distinct metabolic programs (32–34). For example, exposure to Toll-like receptor (TLR) agonists such as bacterial lipoproteins, peptidoglycan, DNA, or lipopolysaccharide (LPS) biases macrophages towards glycolysis. In contrast, anti-inflammatory cytokines (i.e. IL-4 or IL-10) or growth factors promote oxidative phosphorylation (35, 36). In pro-inflammatory macrophages, the increase in glycolytic metabolism following TLR activation is due, in part, to two TCA cycle blocks (**Figure 2**) (15, 35). These break points create an anaplerotic cycle that leads to the progressive accumulation of metabolites such as succinate and aconitate, which are immunomodulatory in nature. Initial experiments showed that the intracellular concentration of succinate dramatically increases following TLR stimulation (37–39). Early work into understanding metabolic reprogramming found that this succinate pool was diverted from normal TCA cycle metabolism and oxidized by succinate dehydrogenase (SDH) to produce high amounts of ROS, which stabilized the transcription factor hypoxia-inducible factor-1α (HIF-1α) by preventing its ubiquitination and degradation (40–44). HIF-1α stabilization leads to the production of pro-IL-1β, which is cleaved into its mature form by the NOD-like receptor pyrin domain containing 3 (NLRP3) inflammasome (45–47). A secondary effect of this process is the HIF-1α-dependent induction of glycolytic enzymes by that feed back to promote biosynthetic pathways to augment pro-inflammatory activity (40, 48, 49). Increased glycolysis serves to produce ATP and sustain the mitochondrial membrane potential that is necessary for succinate oxidation.

In macrophages, NLRP3 inflammasome activation generally occurs *via* a two signal model (45). Signal 1 is mediated by TLR, IL-1R, or TNFR activation that elicits maximal expression of pro-IL-1β and inflammasome components. Numerous stimuli have been shown to provide signal 2 and the diverse structure of these molecules has led to the concept that cellular stress is a unifying factor responsible for NLRP3 inflammasome activation (45). With regard to *S. aureus*, α-toxin acts as signal 2 to activate a primed NLRP3 inflammasome (50). This has been attributed to K^+^ efflux from cells as a consequence of toxin-mediated membrane disruption, which can be potentiated by gasdermin D cleavage by the NLRP3 inflammasome that forms another transmembrane pore leading to pyroptosis (51). However, *in vivo* studies have shown that α-toxin alone is not sufficient for NLRP3 activation. Another requisite is *S. aureus* lipoproteins that provide signal 1 *via* TLR2 activation to elicit maximal expression of pro-IL-1β and inflammasome components (52). Recently, it was shown that *S. aureus* packages its pore-forming toxins into extracellular vesicles that are then internalized by immune cells. Upon uptake, *S. aureus*-derived vesicles contain all of the requisite factors to induce inflammasome activation, thus providing the bacteria with another mechanism to modulate the immune response (53). A paradox is why *S. aureus* augments NLRP3 inflammasome activation given its ability to produce the pro-inflammatory cytokines IL-1β and IL-18. However, a recent study has demonstrated that *S. aureus* α-toxin exploits NLRP3 inflammasome activation in macrophages by recruiting mitochondria away from the phagosome, which inhibits mitochondrial ROS production *via* complex II (SDH) of the electron transport chain (ETC), phagosomal acidification, and bacterial killing (54). This effect was independent of NLRP3-mediated IL-1β and IL-18 production. Intriguingly, this represents another mechanism that *S. aureus* exploits to prevent immune-mediated clearance.

Subsequent *in vitro* studies examining the mechanisms of macrophage metabolic remodeling revealed that the increase in succinate following TLR activation was due to the action of itaconate, a derivative of the TCA intermediate aconitate. Itaconate is produced by immune-responsive gene 1 (IRG1) and as the concentration of itaconate increases, SDH is progressively inhibited. This leads to succinate accumulation and decreased oxygen consumption *via* the inhibition of SDH, which is also complex II of the mitochondrial ETC (**Figure 2**) (55–59). Although the initial production of itaconate augments macrophage pro-inflammatory activity, its accumulation begins to exert anti-inflammatory effects (60). As itaconate accumulates, it is transported out of mitochondria where it can interact with cytoplasmic protein targets, namely Kelch-like ECH-associated protein 1 (KEAP1) (61–63). Under homeostatic conditions, KEAP1 is bound to nuclear factor erythroid 2-related factor 2 (Nrf2), which targets the complex for proteasomal degradation. Under stress conditions, such as *S. aureus*-induced toxin action, the complex dissociates and Nrf2 translocates to the nucleus where it acts as a transcription factor for numerous anti-inflammatory genes (64). Itaconate is capable of disrupting the KEAP1-Nrf2 association *via* alkylation of cysteine residues in KEAP1 to promote Nrf2 nuclear translocation and the transcriptional activation of anti-inflammatory genes. Via this mechanism, itaconate represents a way to counterbalance the proinflammatory activity of succinate accumulation (65, 66).

Amino acid metabolism is also important for influencing macrophage polarization, where arginine is differentially utilized by macrophages to exert distinct effector functions (67). For example, in response to planktonic *S. aureus* and other pro-inflammatory stimuli, macrophages utilize arginine to drive inducible nitric oxide synthase (iNOS) activity and nitric oxide (NO) production (68). Nitric oxide is a highly reactive free radical that exerts potential bactericidal activity by inducing DNA and membrane damage as well as targeting oxidative metabolism (69–71). However, *S. aureus* is capable of evading host NO production, which differentiates it from other Staphylococcal species. One example is flavohemoprotein (*hmp*) expression that allows *S. aureus* to detoxify its environment by converting NO into nitrate, making it an iNOS-dependent virulence determinant (72). Another component of the nitrosative stress response in *S. aureus* is L-lactate dehydrogenase (*ldh1*), which is a NO-inducible gene. While hmp serves to detoxify the environment, Ldh allows *S. aureus* to maintain redox homeostasis by promoting the conversion of pyruvate to lactate (73). Although this metabolic program generates less ATP compared to oxidative metabolism, it provides a mechanism by which *S. aureus* can maintain its reducing equivalents until Hmp can decrease NO levels. In contrast to iNOS, arginine is used by arginase-1 (Arg-1) in anti-inflammatory polarized macrophages to produce ornithine. Ornithine is further metabolized into polyamines and proline for wound repair and cell growth processes (74, 75). Increased *Arg1* expression has been linked to macrophage anti-inflammatory activity during *S. aureus* biofilm infection (76–78). To determine if *Arg1* expression was required for the immune suppression associated with *S. aureus* biofilm infection, our laboratory utilized Arg-1^fl/fl^; Tie-2^Cre^ conditional knockout mice where myeloid cells lacked Arg-1. *Arg1* was dispensable for myeloid immunosuppression during biofilm formation but was critical for *S. aureus* containment during abscess formation (79). This was in agreement with a prior study showing that host polyamine production was important for controlling *S. aureus* growth in a mouse SSTI model (80). Taken together, these results indicate that the effects of *Arg1* expression are context-dependent in terms of myeloid cell function during *S. aureus* biofilm vs. planktonic infection.

Monocyte/macrophage metabolism was recently shown by our laboratory to be important for influencing the outcome of *S. aureus* biofilm infection in a mouse model of PJI (81). *S. aureus* biofilm biased monocytes towards oxidative metabolism which, much like macrophages exposed to biofilm *in vitro*, were largely anti-inflammatory in nature (82). Therefore, a nanoparticle approach was used to deliver oligomycin, an inhibitor of complex V of the ETC, to redirect monocyte metabolism towards glycolysis. Oligomycin nanoparticles augmented monocyte pro-inflammatory activity, which coincided with reduced biofilm burden *in vivo*, indicating that metabolic remodeling could prove to be an effective therapeutic approach for chronic biofilm infection (81). Importantly, monocyte metabolic reprogramming was capable of attenuating an established biofilm infection, whose efficacy was heightened by concominant antibiotic treatment.

## Granulocyte Metabolism

Although much progress has been made in understanding the role of metabolism in macrophages, comparatively less is known about how metabolic changes affect granulocyte function. Neutrophils have been shown to rely almost entirely on glycolytic modes of metabolism to fuel their effector functions, which agrees with a reduced mitochondrial abundance (83, 84). Similar to pro-inflammatory macrophages, activated neutrophils adopt a metabolic program that is similar to the aerobic glycolysis that was first described by Otto Warburg in the 1920’s (85), which has since become known as “Warburg metabolism” (86, 87). This heavy reliance on glycolysis, even in oxygen replete conditions, is necessary to increase carbon flux through the pentose phosphate pathway (PPP) to increase the NADPH pool that is required for NADPH oxidase activity and ROS production (16, 88). Like pro-inflammatory macrophages, metabolic intermediates from the TCA cycle that is fueled by glycolysis, are used for anabolic processes to promote granulocyte functional activity. For example, citrate from the TCA cycle can be diverted for fatty acid synthesis to drive the production of pro-inflammatory mediators such as prostaglandins and leukotrienes (89–92). Although granulocytes are typically considered as purely glycolytic, some studies have also identified granulocytes that instead utilize mitochondrial oxidative metabolism (93). In the context of cancer, glucose within the tumor microenvironment can quickly become a limiting factor. Under these conditions, a population of immature, c-Kit^+^ neutrophils has been shown to utilize fatty acid oxidation to maintain ROS production by NADPH oxidase (93). These immature neutrophils in tumor-bearing mice were regulated through aberrant SCF/c-Kit signaling and metabolically adapted for the lack of glucose within the tumor microenvironment. Functionally, this meant the adapted neutrophils retained the ability to produce ROS, which can interfere with CD4^+^ T cell anti-tumor activities (94–96). Although not definitively established, these c-Kit^+^ neutrophils possess many characteristics of granulocytic MDSCs (G-MDSCs, see below). A similar reduction in glucose availability occurs during *S. aureus* biofilm formation, as reflected by a shift towards fermentative metabolism and lactate production (23), which shapes the metabolic attributes of infiltrating leukocytes, which is discussed below.

MDSCs are a heterogenous population of immature myeloid cells that are grouped into two categories based on their shared characteristics with mature monocytes or neutrophils, namely M-MDSCs and G-/PMN-MDSCs (97–99). Both subsets are thought to exert their suppressive activity through increased ROS production, although M-MDSCs can also utilize Arg-1 to deplete arginine that is required for TCR expression to inhibit T cell activation (94, 100, 101). Although MDSCs have been best characterized in cancer, they have also been implicated in promoting chronic infection, including *S. aureus* biofilm (25–28), bone, and skin infection (102, 103). Due to the extensive heterogeneity of MDSCs and context-dependent modes of action, describing a singular metabolic program that is characteristic of these cells has proved challenging. Nevertheless, a few reports have examined MDSC metabolism and how this affects their suppressive activity (104–106). One study found that M-MDSCs suppress T cell activation by inhibiting glycolysis through direct physical contact. Interestingly, M-MDSCs were metabolically dormant, characterized by an accumulation of the α-dicarbonyl methylglyoxal. Methylglyoxal was found in T cells following co-culture with M-MDSCs and treatment with dimethylbiguanide (DMBG), which neutralizes dicarbonyls, restored T cell activation, supporting the importance of methylglyoxal in MDSC-mediated T cell suppression (107, 108). Another study has pointed to fatty acid accumulation and subsequent prostaglandin production as a metabolic mechanism for MDSC suppression. Specifically, PMN-MDSCs overexpress a fatty acid transporter (FATP2), which led to fatty acid accumulation and prostaglandin E_2_ (PGE_2_) production that promoted their immunosuppressive effects (109). These studies highlight the metabolic diversity that MDSCs can adopt to influence their inhibitory activity. *S. aureus* biofilm elicits G-MDSCs that may utilize a distinct metabolic program to exert their suppressive effects than those described here, which remains to be determined. Further investigation into how MDSC metabolism evolves throughout the course of infection is required to appreciate the role that these cells play in shaping the host immune response.

## Host–Pathogen Metabolic Crosstalk

In an infectious milieu, not only will leukocytes and the pathogen have to compete for the same nutrient sources, but this also creates a new environment in which the two could interact. For instance, a recent study demonstrated how host adapted strains of *P. aeruginosa* responded to the secretion of itaconate by selecting for variants that were able to utilize the host-derived metabolite as a nutrient source (110). This selection process coincided with modifications of membrane structural components in *P. aeruginosa* to augment host-derived itaconate release, thereby establishing a positive feedback loop to promote chronic infection. This illustrates the importance of not only considering the metabolism of either the host or pathogen in isolation, but also the byproducts that they excrete and exchange. Such molecules constitute a new avenue for cellular signaling that the host and/or pathogen could leverage to their benefit during infection. These systems become even more complex in the context of polymicrobial infections, a common occurrence with *P. aeruginosa* and *S. aureus* in the lungs of CF patients (111–113). With the additional layering of another organism, the potential number of molecules and interactions increases exponentially.

## *S. aureus* Metabolism and Competition for Nutrients

Competition for the various nutrient sources in an infectious milieu is probably the most intuitive level by which metabolic crosstalk occurs. In an infection, there are at least two entities (i.e. host and pathogen) racing to consume the same resources, which becomes more complicated in the context of polymicrobial infections. Glucose and oxygen are among the first resources that become restricted in these settings and their deprivation can be enough to bias the actions of responding immune cells or pathogens alike. These events can also dictate the nature of immune cell death, which has been reported to occur *via* two types of programmed necrosis, namely necroptosis or pyroptosis. Necroptosis is induced by the interaction of TNF with receptor-interacting protein kinase 1 (RIPK1), eliciting a cascade that culminates in the phosphorylation of mixed lineage kinase domain-like protein (MLKL) that damages the plasma membrane leading to cell death. Pyroptosis occurs in response to inflammasome activation that cleaves gasdermin D, which oligomerizes to form pores in the cell membrane. While both forms of cell death induce inflammation, the heightened production of inflammatory mediators associated with inflammasome activation makes pyroptosis more inflammatory in nature than necroptosis (114). The roles of *S. aureus* metabolism in dictating different modes of immune cell death will be discussed below.

## Carbohydrate Availability and *S. aureus* Metabolism

Much work has been done to elucidate *S. aureus* metabolic adaptations under a variety of *in vitro* and *in vivo* conditions and the reader is directed to several excellent and comprehensive reviews on the topic (9, 23, 24), since only a brief overview is provided here. *S. aureus* utilizes a number of two-component regulatory systems to sense changes in its environment (115, 116). These systems interface with complex transcriptional networks to tightly control nutrient use throughout different phases of growth and infection. This is referred to as carbon catabolite repression and ensures that bacteria optimally utilize available nutrient resources in a hierarchical manner (117–119). *S. aureus* uses two catabolite control proteins (CcpA & CcpE) to modulate glucose utilization through central carbon metabolism. In glucose replete conditions, oxidative metabolism is repressed by CcpA, which inhibits the expression of critical TCA cycle enzymes (120–122). As glucose becomes depleted, CcpA activity is reduced which induces the TCA cycle and citrate production. Citrate is sensed by CcpE, which bolsters TCA cycle activity and increases the expression of *S. aureus* virulence factors (123, 124). It has been shown that *S. aureus* can quickly outcompete host keratinocytes for available glucose, which leads to keratinocyte death by pyroptosis (125, 126). Pyroptosis fails to clear *S. aureus* infection because NLPR3 inflammasome activation by α-hemolysin redirects mitochondria away from bacteria-containing phagosomes, thereby preventing acidification and killing (54). Thus, pyroptosis is beneficial for the bacteria because it promotes its intracellular escape and dissemination, while simultaneously introducing any host-derived metabolic intermediates into the infection milieu to be utilized for pathogen survival and replication.

The importance of this battle for glucose is particularly relevant in the setting of diabetes. Diabetic patients are characterized by hyperglycemia and often present with persistent and invasive *S. aureus* infections (127, 128). A recent study examining *S. aureus* SSTI in a diabetic mouse model has shown that although there is more bioavailable glucose in diabetic tissues, phagocytes failed to take up the carbohydrate with either GLUT-1 or GLUT-3 transporters. This resulted in impaired oxidative burst activity and increased bacterial burden (129). *S. aureus* infection in diabetic patients has been linked to an increase in the recruitment of low-density neutrophils (LDNs) (130). LDNs are associated with increased rates of NETosis, or the production of neutrophil extracellular traps (NETs). Although, NETs are typically considered to exert anti-microbial effects, elevated levels of NETosis have been linked to impaired wound healing in patients with diabetes, whose neutrophils are more prone to NET formation (131). Increased NETosis in diabetic patients could result from the elevated levels of glucose within diabetic tissues, as glucose is a metabolic requirement for the process. Of note, MDSCs that are expanded in the blood of tumor patients have been characterized as LDNs (132, 133). By extension, it is intruging to speculate that the LDNs described in diabetic patients are actually MDSCs, which might explain why these individuals are more prone to chronic and recurrent *S. aureus* infections, although this remains to be determined.

## Hypoxia and *S. aureus* Metabolism

Hypoxia is particularly relevant in the context of biofilm infections, since bacteria in the innermost regions of the biofilm experience low oxygen bioavailability. Anaerobic conditions have been shown to induce the expression of biofilm-associated genes such as *icaADBC* that encode polysaccharide intercellular adhesins that promote bacterial aggregation and adherence to host surfaces (134–136). Bone, a niche that is often targeted by *S. aureus* biofilm, has a low oxygen tension since it is less vascularized compared to other tissues (137, 138). The immune response elicited during *S. aureus* bone infection exacerbates this hypoxic environment as infiltrating immune cells quickly increase their oxygen demand upon activation (139, 140). The biofilm responds to this progressive decline in oxygen tension by switching from respiration to fermentation concomitant with an increase in virulence factor production to attack immune cells to promote infection persistence (141). The transition from respiration to fermentation in *S. aureus* is regulated by several factors including SrrAB and Rex. The SrrAB two-component system was initially identified through homology comparisons with the ResDE two-component system of *Bacillus subtilis*, which controls the switch between aerobic and anaerobic respiration (142). While its ligand remains unknown, SrrAB increases the expression of fermentative genes such as *pflAB*, *adhE*, and *nrdDG* that allow it to thrive in an anaerobic environment (141, 143, 144). *S*. *aureus* can also respond to changes in oxygen availability indirectly through the transcriptional repressor Rex, which inhibits genes that are important for anaerobic respiration when oxygen is present. Rex senses redox conditions within the cell through changes in the NADH/NAD^+^ pools. As NADH levels rise, Rex becomes derepressed and is released from the DNA, thereby allowing for the transcription of fermentative genes in an effort to maintain reducing equivalents within the cell (9, 145). Therefore, oxygen depletion could either be a consequence of bacterial growth, or a strategy enacted by the biofilm to ensure persistent infection.

## Epigenetic Changes Induced by Immunomodulatory Metabolites

**Figure 3:**
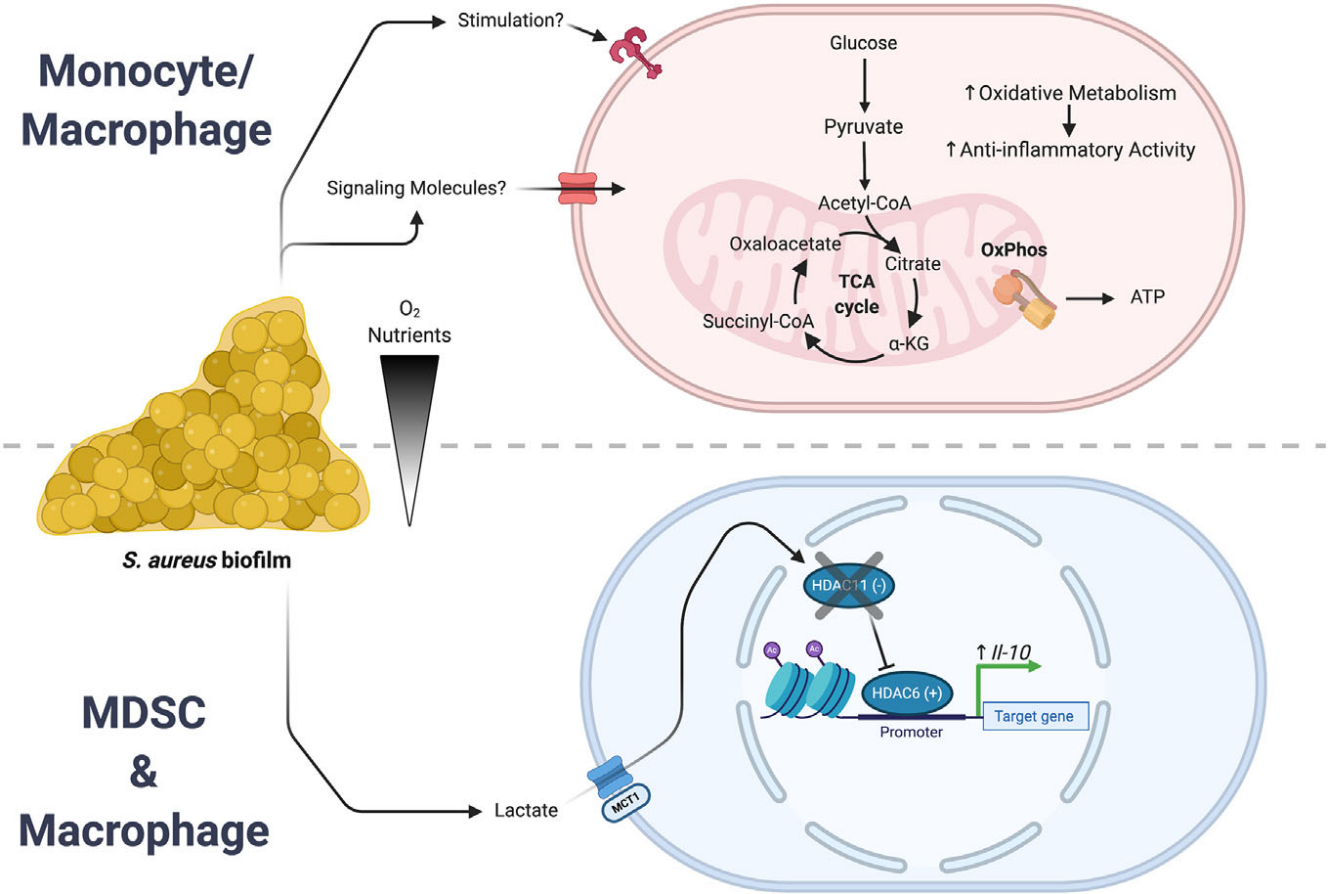
*S. aureus* biofilm regulates leukocyte inflammatory activity. *S. aureus* biofilms employ a number of strategies to create an infection milieu to ensure persistence. Two of these approaches involve the action of either bacterial-derived metabolites or intrinsic reprogramming of monocyte/macrophage metabolism that culminate in the expression of anti-inflammatory genes. (Top) Biofilm augments oxidative metabolism in infiltrating monocytes and macrophages that biases them towards an anti-inflammatory phenotype. (Bottom) In MDSCs and macrophages, biofilm-derived lactate causes epigenomic remodeling that leads to an increase in IL-10 expression. Figure created with BioRender.

Bacterial-derived metabolites are also capable of affecting leukocyte activation and function. Recent work has shown that lactate production by *S. aureus* biofilm induces epigenetic changes in leukocytes at the level of histone acetylation (146). Utilizing a number of *S. aureus* lactate dehydrogenase (*ldh*) mutants, our group demonstrated that biofilm-derived lactate was imported into MDSCs and macrophages where it inhibited histone deacetylase 11 (HDAC11; **Figure 3**). HDAC11 inhibition prevented its normal function of counterbalancing HDAC6 activity that is a positive regulator of IL-10 transcription, resulting in enhanced IL-10 production and biofilm persistence. Synovial fluid from patients with PJI contained elevated amounts of both D-lactate and IL-10 compared with control subjects and IL-10 production by human monocyte-derived macrophages was induced by biofilm-derived lactate, supporting the translational relevance of these findings (146). This demonstrates how a bacterial-derived metabolite can significantly rewire the host-pathogen dynamic to favor persistent infection. Recently, lactate has also been shown to directly modify histones with functional genomic implications (147–149). This lactate modification of histone lysine residues (termed lactylation) was shown to operate as a sort of “clock”. As inflammation progressed, the lactate produced by increased glycolysis lactylated histones. As these lactate marks accumulated, homeostatic genes were induced that led to a resolution of inflammation. Thus, histone lactylation functions as an endogenous timer for inflammatory events.

**Figure 4:**
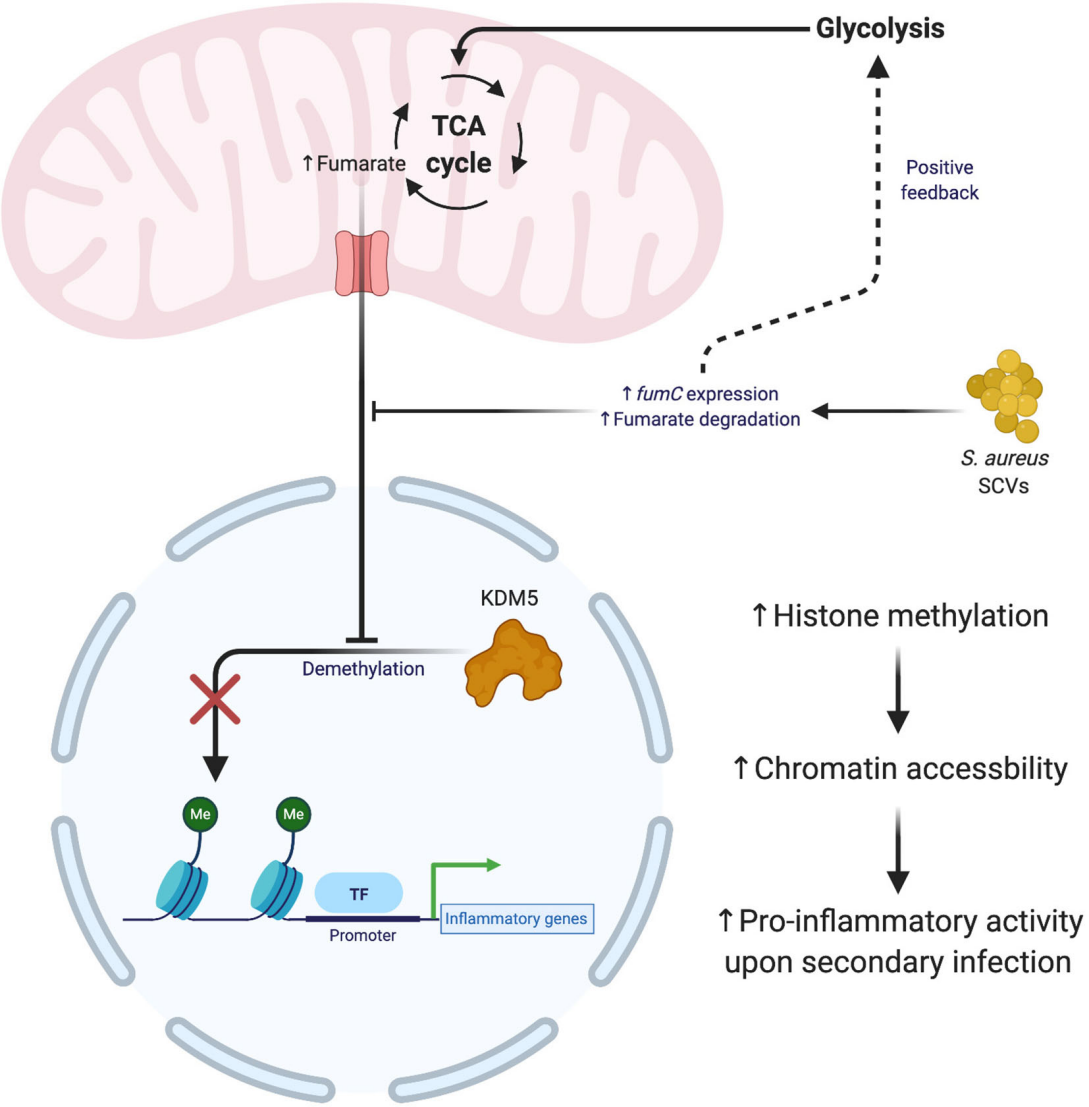
*S. aureus* small colony variants (SCVs) interfere with the establishment of trained immunity. The generation of SCVs with mutations in metabolic pathways exert influence over the formation of trained immunity. *S. aureus* SCVs with hyperactive fumarate hydratase (fumC) quickly degrade mitochondrial pools of fumarate, which leads to an upregulation of glycolysis and impaired formation of trained immunity. The absence of fumuarate causes KDM5 to become active, which removes the methylation marks around the promoters of pro-inflammatory genes, thereby decreasing the accessilibity of the chromatin in these regions. Figure created with BioRender.

While innate immune cells do not possess the characteristic long-lasting immunity of lymphocytes, they are capable of trained immune memory (150, 151). Trained immunity arises from epigenetic modifications that prime leukocytes for a subsequent encounter with another stimulus (152–154). Interestingly, trained immunity is not stimulus-specific and can induce widespread changes in how a leukocyte responds to a second insult. Several studies have shown that prior infection with *S. aureus* establishes trained immunity in macrophages that provides temporary protection from a second bacterial challenge (155–157). Trained immunity in response to *S. aureus* has been shown to occur *via* epigenetic changes induced by the metabolite fumarate. Fumarate, like lactate, interferes with the epigenomic remodeling of histone marks by acting as an antagonist for lysine demethylases (KDMs) (158–162). A recent study demonstrated the importance of fumarate during *S. aureus* infection by contrasting wild type infection with Δ*hemB* small colony variants (SCVs) that were able to deplete local fumarate stores *via* enhanced fumarate hydratase (*fumC*) expression (163, 164). Trained immunity was assessed by comparing bacterial burden after a secondary challenge with *S. aureus* 28 days following the primary infection. The reduction in fumarate by SCVs increased host cell glycolysis, which inhibited trained immunity by necroptosis (**Figure 4**) rather than inflammasome-dependent pyroptosis (165). This is beneficial for bacterial persistence since necroptosis elicits less inflammation compared to pyroptosis (166). These effects were not observed with wild type *S. aureus*, which was less effective at utilizing fumarate. This difference may represent one explanation for why *S. aureus* SCVs are typically associated with chronic infections.

## Modulating Metabolism as a Therapeutic Target

The selective targeting of either host or pathogen metabolism represents an exciting therapeutic prospect that would bolster traditional antibiotic therapies that are commonly used for infections. Metabolic interventions have the potential to be highly efficacious as was shown in our work where metabolically reprogramming monocytes synergized with antibiotics to reduce established *S. aureus* biofilm infection to below the limit of detection (81). Of particular interest are therapies targeting immunometabolism rather than bacterial metabolism, since pathogen resistance would be less prevalent with a host-targeted approach. If we can gain a better understanding of the underlying metabolic modules that dictate beneficial vs. detrimental immune responses, then it should be feasible to tailor leukocyte metabolism to enhance pathogen neutralization. The main caveat of this approach is that the metabolic pathways utilized by the host and pathogens share many attributes. Therefore, potential metabolic therapies would need to be targeted to avoid non-specific effects.

## Conclusion and Future Directions

In a time of increasing interest in leukocyte metabolism, it is important to think globally in terms of the interplay between host and pathogen metabolic states. Bacterial pathogens, such as *S. aureus*, undergo their own metabolic programming (10, 11), often in direct competition with responding immune cells. In addition, the metabolic attributes of tissue resident parenchymal cells have the potential to shape the metabolic responses of both infiltrating leukocytes and bacteria, revealing another level of complexity. Considerable care must be taken when designing experiments to deconstruct these complex systems *in vitro*, since the composition of mammalian cell culture media can have drastic deviations from metabolite concentrations found in human plasma (167). Therefore, findings must be validated in leukocytes and bacteria immediately *ex vivo*, as the selection of *in vitro* culture conditions could result in metabolic changes that are not reflective of *in vivo* infection (167).

As discussed in this review, *S. aureus* as well as other bacterial pathogens possess the ability to not only modify their metabolism, but also that of the host. Further work is needed to understand the numerous intricacies that underpin metabolism, especially in the context of an infection where multiple players are involved. Fortunately, the field is advancing rapidly with the development of novel tools and methods to dissect metabolism with single cell resolution (168, 169). The continued development of these technologies will be integral for future studies. Should these efforts be successful, metabolic modulation could advance therapeutic approaches for *S. aureus* infection in combination with conventional antibiotic treatment regimens.

## Author Contributions

CH wrote the manuscript draft that was edited by TK and CH. All authors contributed to the article and approved the submitted version.

## Funding

The Kielian laboratory is supported by NIH grants R01 NS107369 and 3P01AI083211 (Project 4 to TK).

## Conflict of Interest

The authors declare that the research was conducted in the absence of any commercial or financial relationships that could be construed as a potential conflict of interest.
